# Identification of FOXM1 as a therapeutic target in B-cell lineage acute lymphoblastic leukaemia

**DOI:** 10.1038/ncomms7471

**Published:** 2015-03-10

**Authors:** Maike Buchner, Eugene Park, Huimin Geng, Lars Klemm, Johanna Flach, Emmanuelle Passegué, Hilde Schjerven, Ari Melnick, Elisabeth Paietta, Dragana Kopanja, Pradip Raychaudhuri, Markus Müschen

**Affiliations:** 1 Department of Laboratory Medicine, University of California San Francisco, San Francisco, California 94143, USA; 2 Department of Haematology, University of Cambridge, Cambridge CB2 OAH, UK; 3 The Eli and Edythe Broad Center for Regenerative Medicine and Stem Cell Research, Department of Medicine, Hem/Onc Division, University of California San Francisco, San Francisco, California 94143, USA; 4 Division of Hematology and Oncology, Weill Cornell Medical College, New York, New York 10021, USA; 5 Montefiore Medical Center, Albert Einstein College of Medicine, Bronx, New York 10466, USA; 6 Department of Biochemistry and Molecular Genetics, University of Illinois at Chicago, Chicago, Illinois 60607, USA

## Abstract

Despite recent advances in the cure rate of acute lymphoblastic leukaemia (ALL), the prognosis for patients with relapsed ALL remains poor. Here we identify FOXM1 as a candidate responsible for an aggressive clinical course. We show that FOXM1 levels peak at the pre-B-cell receptor checkpoint but are dispensable for normal B-cell development. Compared with normal B-cell populations, FOXM1 levels are 2- to 60-fold higher in ALL cells and are predictive of poor outcome in ALL patients. FOXM1 is negatively regulated by FOXO3A, supports cell survival, drug resistance, colony formation and proliferation *in vitro*, and promotes leukemogenesis *in vivo*. Two complementary approaches of pharmacological FOXM1 inhibition—(i) *FOXM1* transcriptional inactivation using the thiazole antibiotic thiostrepton and (ii) an FOXM1 inhibiting ARF-derived peptide—recapitulate the findings of genetic FOXM1 deletion. Taken together, our data identify FOXM1 as a novel therapeutic target, and demonstrate feasibility of FOXM1 inhibition in ALL.

## Abstract (web summary)

FOXM1, a transcription factor with roles in cell cycle progression, is highly expressed in the majority of solid tumours. Here the authors show that FOXM1 is an ideal therapeutic target in B-cell acute lymphoblastic leukaemia (ALL) due to its dispensability for normal B-cell development.

## Graphical abstract

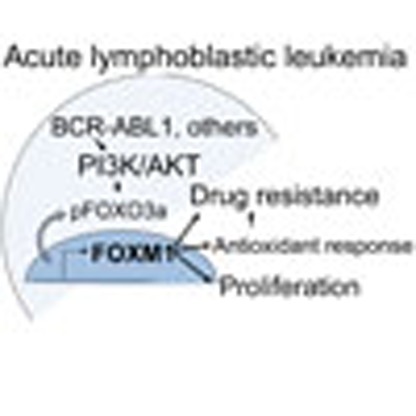

FOXM1 belongs to the forkhead box transcription factor family and is a key regulator of cell growth by promoting cell cycle progression^1^. Expression of the FOXM1 protein is low in quiescent cells. During re-entry into the cell cycle, FOXM1 is expressed at late G_1_/early S-phase, sustained throughout the G_2_ phase and mitosis and its activity is regulated via phosphorylation^2^,^3^,^4^. This phosphorylation relieves it from its autoinhibitory conformation and allows it to drive the expression of additional cell cycle promoting molecules, such as Cdc25A as well as Skp2 and Cks1 (refs 5, 6) FOXM1 expression levels remain elevated in the G_2_- and M-phase, inducing the transcription of cyclin B1 (*CCNB1*), Aurora B kinase (*AURKB*) and Polo-like kinase 1 (*PLK1*) that are responsible for mitotic progression, spindle formation and chromosome segregation. Besides its role in cell cycle progression, FOXM1 has also been described to support cell survival through regulation of the antioxidant defense machinery of the cell, for example, by upregulating superoxide dismutase and Catalase (Cat) expression^7^.

Overexpression of FOXM1 has been implicated in the progression and drug resistance in a wide range of solid tumours^8^ including liver^9^,^10^, colon^11^, lung^12^,^13^,^14^ and prostate carcinoma^15^. Several studies highlight the function of FOXM1 in promoting cell proliferation, migration, angiogenesis and drug resistance that contributes to tumour initiation, growth and progression using transgenic mice as well as FOXM1 inhibitors^8^,^9^,^10^,^11^,^12^,^13^,^14^,^15^,^16^,^17^,^18^,^19^,^20^,^21^. Inducible deletion of *Foxm1* in mouse models for lung adenomas, colon adenocarcinomas and hepatocellular carcinoma resulted in a significant reduction in tumorigenic potential and cancer cell proliferation^10^,^11^,^12^,^13^,^14^. A functional role of FOXM1 in haematopoietic malignancies has been suggested but further experimental validation is required for understanding the mechanism underlying its expression and contribution to disease progression^16^.

Despite advances in the cure rate of childhood pre-B acute lymphoblastic leukaemia (ALL), the prognosis in older patients and for patients who experienced ALL relapse remains poor^22^. Philadelphia chromosome-positive (*Ph*^*+*^) ALL cells are driven by the oncogenic kinase BCR-ABL1 that arises from the t(9;22)(q34;q11) chromosomal translocation in pre-B cells. This subset has been associated with adult ALL and has a particularly poor clinical outcome^23^,^24^,^25^. The *BCR-ABL1* kinase can be specifically targeted by small-molecule tyrosine kinase inhibitors (TKIs) such as imatinib^26^. However, in contrast to *BCR-ABL1*-driven chronic myeloid leukaemia (CML), *Ph*^*+*^ ALL patients will invariably relapse after a short interval of remission, and develop TKI-resistant disease^27^.

Pre-B ALL emerges in virtually all cases from B-cell precursors that are arrested at the pre-B-cell receptor checkpoint. In a gene expression survey of early B-cell development, we found specific upregulation of FOXM1 at the pre-B-cell receptor checkpoint (Fig. 1a). Therefore, we investigate here the function of FOXM1 in normal B-cell development and in pre-B-cell-derived ALL with specific focus on its regulation and function in *Ph*^*+*^ ALL. We reveal a FOXO3a-mediated transcriptional control of FOXM1 expression, a crucial function of FOXM1 with respect to TKI resistance and disease progression, using a conditional *Foxm1*^fl/fl^ knockout mouse model as well as pharmacological inhibition of FOXM1 in patient-derived pre-B ALL cells.

## Results

### FOXM1 expression is dispensable in B-cell precursors

**Figure 1:**
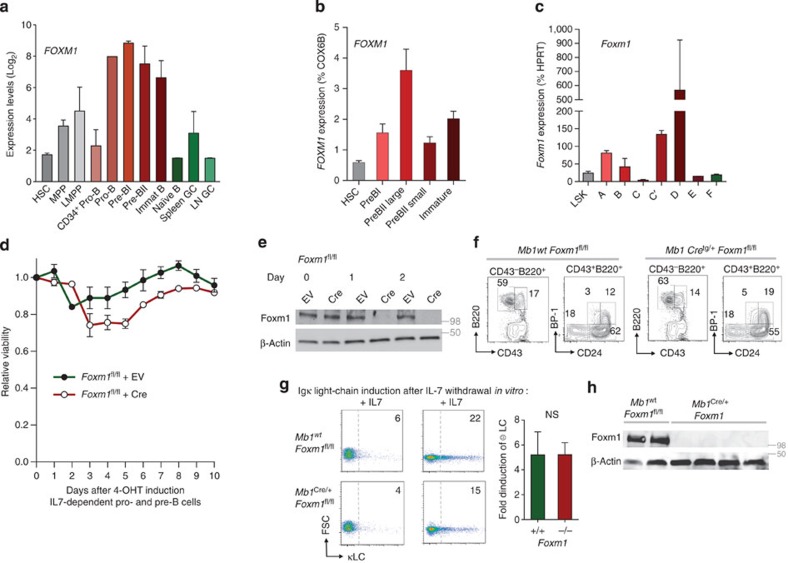
FOXM1 expression is dispensable for normal B-cell survival and development. (**a**) *FOXM1* mRNA expression in sorted progenitor and B-cell fractions according to microarray data^28^ (**b**) qRT–PCR of FOXM1 in human B-cell progenitor fractions with *COX6B* used as a reference, mean values of *n*=3 (technical replicates); ±s.e.m. (**c**) qRT–PCR of Foxm1 in Hardy B-cell fractions is shown with *Hprt* used as a reference, *n*=3 (technical replicates); ±s.e.m.; representative example of two independent BM sorts. (**d**) Viability of *Foxm1*^fl/fl^ B-cell precursors was analysed daily after induction of deletion by 4-OHT; representative results of three independent experiments are shown. (**e**) Immunoblot of Foxm1 deletion induced by 4-OHT in *Foxm1*^fl/fl^ B-cell precursors. (**f**) Representative examples of Hardy Fraction A–C′ for wt BM and *Mb1-Cre*^tg/+^ *Foxm1*^fl/fl^ are shown. (**g**) BM of *Mb1-Cre*^tg/+^ *Foxm1*^fl/fl^ (*n*=4) and *Mb1* wt *Foxm1*^fl/fl^ (*n*=3) littermates were cultured in the presence of IL-7. κ LC expression was induced by IL-7 withdrawal and analysed by flow cytometry after 48 h, statistical significance was tested by Student’s *t*-test. (**h**) IL-7 cultures *Mb1* wt (*n*=2) and from *Mb1-Cre*^tg/+^ *Foxm1*^fl/fl^ (*n*=4) littermates were analysed for Foxm1 expression by immunoblot to confirm *Cre*-mediated *Foxm1* deletion.

We found FOXM1 mRNA specifically upregulated at the pre-B-cell receptor checkpoint (Fig. 1a)^28^. This was verified by quantitative real-time (qRT) PCR of sorted human B-cell precursors as well as murine B-cell progenitor fractions (Fig. 1b,c; sorting strategies and reanalysis of the sort are shown in Supplementary Fig. 1)^28^,^29^. To determine a potential function of FOXM1 in normal B lymphopoiesis, we harvested bone marrow (BM) of a *Foxm1* conditional knockout mouse model (*Foxm1*^fl/fl^)^20^. B-cell precursors of *Foxm1*^fl/fl^ mice were cultured in interleukin 7 (IL-7)-containing media and transduced with a 4-hydroxy tamoxifen (4-OHT)-inducible Cre-ER^T2^ (Cre) or empty vector (EV; ER^T2^). 4-OHT-mediated deletion of *Foxm1* did not significantly alter the viability of normal B-cell precursors (Fig. 1d,e, respectively) and is therefore not required for survival of IL-7-dependent pro/pre-B cells. Next we sought to analyse a potential role of Foxm1 *in vivo* during normal B-cell development. To this end, we crossed *Foxm1*^fl/fl^ mice with *Mb1-Cre*^tg/+^ mice to induce deletion of *Foxm1* in early B-cell progenitors^30^. BM from 6–8-week-old *Mb1-Cre*^tg/+^ *Foxm1*^fl/fl^ mice or *Mb1*^+/+^ wt (wild-type) *Foxm1*^fl/fl^ littermates were analysed for Hardy fractions A–F^29^. In addition, spleen, lymph nodes and peritoneal cavity were analysed for number of B cells and their immunoglobulin light-chain expression (κ and λ). Despite the observed high expression of Foxm1 mRNA in the C′ and D fractions, *Foxm1* deletion did not alter B-cell development (examples of flow cytometry plots are shown in Fig. 1f, further analysis is shown in Supplementary Fig. 2a–d). Also the ability of pre-B cells to differentiate into κ-light-chain producing immature B cells *in vitro* was not affected by B-cell-specific deletion of *Foxm1* (Fig. 1g). The confirmation of *Foxm1* deletion is shown by immunoblot in Fig. 1h.

To further define whether Foxm1 expression is required for the proliferation and survival of uncommitted cells, we isolated BM cells from *Foxm1*^fl/fl^ mice and cultured them in the presence of IL-3/IL-6/SCF to induce proliferation of Lin^−^ Sca-1^+^ c-Kit^+^ (LSK)-like haematopoietic progenitor cells and transduced them with EV or inducible Cre-green fluorescent protein (GFP)^31^. We followed GFP expression and observed no change of growth behaviour on Foxm1 deletion in LSK progenitor cells (Supplementary Fig. 2e).

### FOXM1 is highly expressed in ALL

**Figure 2:**
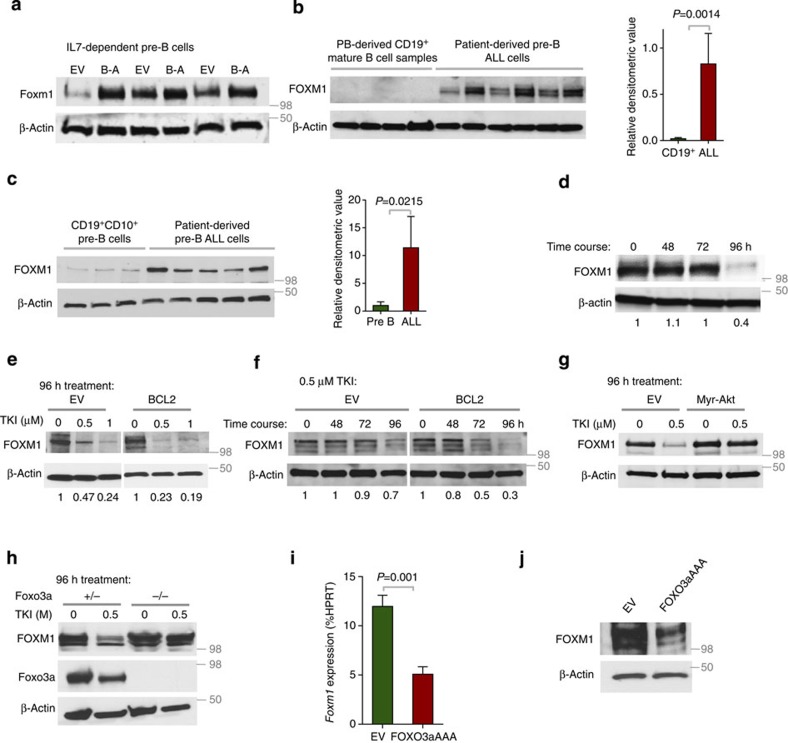
Regulation of FOXM1 expression in patient-derived pre-B ALL cells. (**a**) Foxm1 expression levels of three matched samples of IL-7-dependent B-cell precursors (in the presence of IL-7) and BCR-ABL1-transformed ALL-like cells (IL-7 independent). (**b**,**c**) Immunoblot analysis for the expression of FOXM1 in normal CD19^+^ B cells (**b**) derived from peripheral blood (PB) or CD19^+^ CD10^+^ B-cell precursors (**c**) derived from BM and patient-derived pre-B ALL; loading order for ALL: ICN1 (*Ph*^*+*^), PDX2 (*Ph*^*+*^), SFO5 (normal karyotype), LAX7 (normal karyotype), LAX7R (normal karyotype), LAX2 (*Ph*^*+*^) and PDX2 (*Ph*^*+*^), MPX2 (*Ph*^*+*^), MPX3 (*Ph*^*+*^), MPX5 (*Ph*^*+*^), BLQ5 (*Ph*^*+*^), respectively; shown with β-actin as loading control. Mean densitometric values relative to loading control are shown in the bar graphs ±s.e.m., significance was determined by Student’s *t*-test. (**d**) Time course of FOXM1 protein levels after TKI treatment (shown for ICN1), β-actin was used as a loading control; representative result of three independent experiments is shown. (**e**) Human *Ph*^*+*^ ALL cells (PDX2) were treated with TKI at low doses for 96 h and analysed for FOXM1 expression with β-actin as a loading control with and without overexpression of BCL2, representative result of three independent experiments is shown. (**f**) Time course analysis of FOXM1 expression in PDX2 with either empty vector or BCL2 overexpression. (**g**) Immunoblot analysis of Foxm1 with and without the expression of constitutively active Myr-Akt in murine ALL, treated with low concentrations of TKI or control for 96 h. (**h**) Immunoblot analysis Foxm1 expression in murine BCR-ABL1-transformed Foxo3a^+/−^ and Foxo3a^−/−^ ALL treated with low concentrations of TKI or control for 4 days; representative result of three independent experiments is shown. (**i**) *Foxm1* qRT–PCR was performed on day 2 after transduction with EV or a constitutively active form of FOXO3a (FOXO3aAAA), *Hprt* was used as a reference, *n*=3; ±s.e.m., Student’s *t*-test was used for statistical analysis. (**j**) Foxm1 protein levels were measured 3 days after the introduction of a constitutively active form of FOXO3a (FOXO3aAAA), with β-actin as a loading control.

To investigate a potential role of FOXM1 in transformed B cells, we used a mouse model for *Ph*^*+*^ ALL: to this end, BM-derived B-cell precursors were cultured in the presence of IL-7 and transformed with a retroviral BCR-ABL1 expression vector (schematic shown in Supplementary Fig. 3a). *De novo* expression of BCR-ABL1 increased levels of Foxm1 compared to normal IL-7-dependent pre-B cells (Fig. 2a). We compared the expression levels in human B-cell populations isolated from BM or peripheral blood of healthy donors with patient-derived pre-B ALL samples. All patient-derived samples used in this study are listed in Supplementary Table 1 and enrichment efficiency of CD19^+^ and CD19^+^CD10^+^ B-cell populations is shown in Supplementary Fig. 3b,c. While FOXM1 protein expression levels were low in both BM-derived B-cell precursors and mature B cells, patient-derived pre-B ALL samples revealed 2- to 60-fold higher FOXM1 protein levels compared with B cells or B-cell precursor populations (*P=*0.0014 and *P*=0.0215, respectively; Student’s *t*-test; Fig. 2b,c). To define whether high FOXM1 expression was specific for defined ALL subsets, we compared the protein levels in *Ph*^*+*^ ALL samples and samples driven by other oncogenes, derived from childhood or adult ALL without observing significant differences (Supplementary Fig. 3d). To further define whether FOXM1 expression was induced by BCR-ABL1 kinase activity, we treated patient-derived *Ph*^*+*^ ALL samples with TKI. Although we did not observe short-term effects, after 96 h of TKI treatment, FOXM1 levels were significantly downregulated (Fig. 2d). To ensure that the observed FOXM1 downregulation is not secondary to apoptosis induction but occurs as a consequence of BCR-ABL kinase inhibition, we overexpressed BCL2 in *Ph*^*+*^ ALL cells and thereby abrogated apoptosis induction by TKI^32^. Similar to EV control cells, TKI treatment induced significant FOXM1 downregulation in the absence of apoptosis induction (Fig. 2e; corresponding viability is shown in Supplementary Fig. 3e). Interestingly, mitigating the effects of selection pressure (via BCL2 expression; Supplementary Fig. 3e) revealed a more rapid and even more profound downregulation of FOXM1 (Fig. 2e,f).

To further characterize the regulation of FOXM1, we analysed whether the inactivation of FOXO factors downstream of the PI3K/AKT pathway contributes to high FOXM1 expression in *Ph*^*+*^ ALL^33^,^34^,^35^,^36^. We overexpressed a constitutively active form of Akt (Myr-Akt) to prevent activation of FOXO factors in the presence of TKI. While this resulted in modest induction of Foxm1 expression in untreated cells, Myr-Akt expression entirely abrogated TKI-mediated downregulation of Foxm1 (Fig. 2g). To define whether Foxo3a is required for Foxm1 downregulation in ALL cells^34^, we generated Foxo3a-knockout *BCR-ABL1*-driven ALL and performed TKI treatment. Strikingly, in the absence of Foxo3a, FOXM1 levels remained high despite long-term TKI treatment (Fig. 2h). To further confirm the regulation of FOXM1 by FOXO3a, we overexpressed a constitutively active form of FOXO3a (FOXO3aAAA)^37^ and observed significant downregulation of Foxm1 mRNA and protein levels compared with empty vector control (Fig. 2i,j). Active FOXO1, however, did not modulate FOXM1 levels on mRNA or protein levels (Supplementary Fig. 3f,g).

### FOXM1 is a predictor of poor clinical outcome in ALL

**Figure 3:**
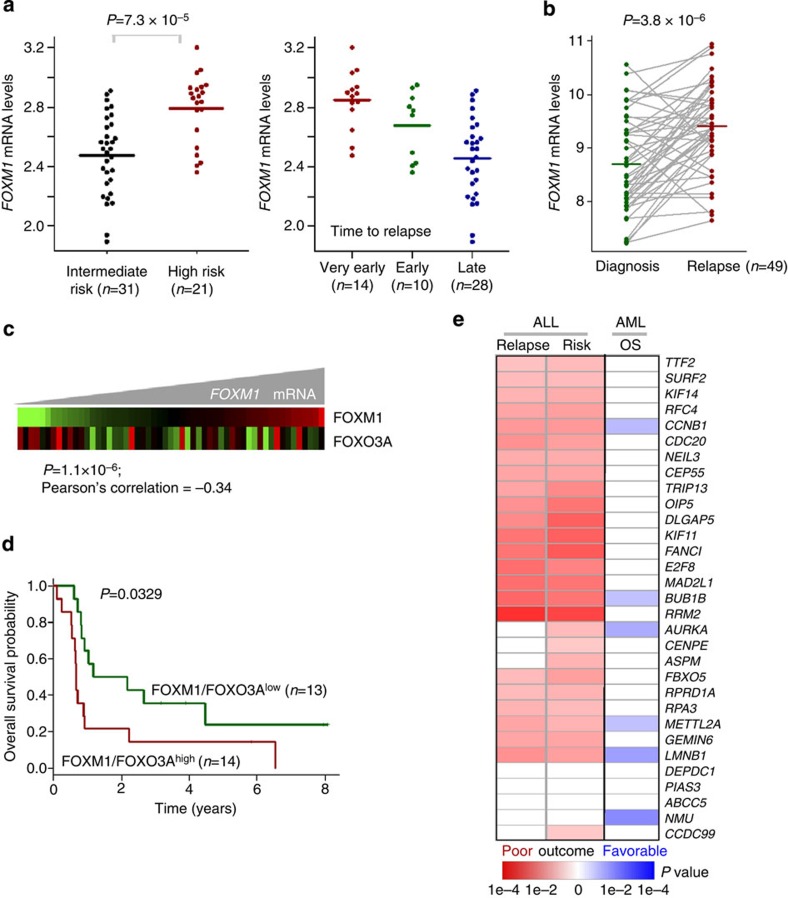
FOXM1 is a predictor of poor clinical outcome in pre-B ALL. (**a**) Gene expression profiling of 60 cases of first relapse childhood ALL samples were analysed for FOXM1 expression and stratified into intermediate and high-risk groups according to a combination of prognostic factors of the relapse trial of the German Berlin–Frankfurt–Münster (BFM) study group ALL-REZ BFM 2002 (left panel; Wilcoxon rank-sum test was used for statistical analysis). Analysis of the same data set was correlated with time to relapse: very early relapse (within 18 months after initial diagnosis; *n*=14), early relapse (>18 months after initial diagnosis but <6 months after cessation of frontline treatment; *n*=10) and late relapse (>6 months after cessation of frontline treatment; *n*=28; right panel; Kruskal–Wallis rank-sum test was used for statistical analysis). (**b**) FOXM1 expression as determined by the gene expression profiling of matched samples of ALL at time of diagnosis and relapse. (**c**) Inverse correlation of FOXM1 with FOXO1A and FOXO3A mRNA levels in patients with *Ph*^*+*^ ALL in the ECOG E2993 trial (*n*=55; Pearson correlation test). (**d**) Patients with *Ph*^*+*^ ALL in the ECOG E2993 trial (*n*=55) were segregated into two groups based on higher or lower than median ratio of mRNA levels of FOXM1/FOXO3A. OS of patients in the FOXM1/FOXO3A^Hi^ versus FOXM1/FOXO3A^Low^ group was compared by Kaplan–Meier analysis with the *P* value determined by Log-rank Mantle–Cox test. (**e**) FOXM1 target genes^18^ on a data set collected by the German ALL-REZ BFM 2002 of the BFM study group (*n*=60)^60^ and AML (data retrieved from The Cancer Genome Atlas). A colour scale indicates whether the expression level of a gene is significantly associated with poor (in red colour) or favourable (in blue colour) patient outcome or not significant (in white colour), based on the parameters of relapse, risk stratification (as determined by Wilcoxon rank-sum test) and OS (as determined by Log-rank Mantle–Cox test).

To evaluate a potential predictive value of FOXM1 mRNA levels in patient-derived ALL samples at the time of diagnosis, we conducted a retrospective analysis of FOXM1 expression. First, we measured FOXM1 mRNA levels at the time of diagnosis in a clinical trial for patients with childhood ALL (BFM-REZ 2002). FOXM1 mRNA levels clearly correlated with risk stratification of childhood ALL and were significantly lower in BM biopsies from patients with intermediate-risk pediatric ALL (*n*=31; including one patient with *Ph*^*+*^ ALL) compared with high-risk pediatric ALL (*n*=21; including one patient with *Ph*^*+*^ ALL; *P*=7.3e−5, Wilcoxon rank-sum test). As a metric for ALL aggressiveness, FOXM1 mRNA expression levels also correlated with time to relapse (Fig. 3a; *n*=52; *P*=0.00015; Kruskal–Wallis rank-sum test). Consistent with data from the BFM-REZ 2002 trial, we found that FOXM1 mRNA levels are upregulated at the time of relapse in a pediatric high-risk ALL trial (COG P9906). Comparative analysis of FOXM1 mRNA levels from matched samples from 49 ALL patients at diagnosis and subsequent relapse revealed a significant upregulation of FOXM1 by the time of relapse (*P*=3.8e−6; paired *t*-test; Fig. 3b). In addition, we found a significant inverse correlation of FOXM1 with FOXO3A mRNA levels in *Ph*^*+*^ ALL patients (Fig. 3c; Pearson correlation test) from the Medical Research Council UKALL XII/Eastern Cooperative Oncology Group (ECOG) E2993 trial. Overall survival (OS) analysis of this patient cohort revealed that a high ratio of FOXM1/FOXO3A was a predictor of poor OS in the high-risk subgroup of *Ph*^*+*^ ALL (Fig. 3d; *n*=27; *P*=0.03; Log-rank Mantle–Cox test). Analyses for the individual genes are shown in Supplementary Fig. 3h,i. Furthermore, a set of genes that is directly regulated by FOXM1 and associated with poor prognosis in breast cancer^18^ showed significant correlation with poor clinical outcome in B-cell lineage ALL patients but not in myeloid lineage AML (Fig. 3e). In summary, these findings indicate that FOXM1 mRNA expression levels are associated with poor clinical outcome in ALL and inversely correlate with mRNA of FOXO3a.

### Foxm1 mediates proliferation and survival of leukaemia cells

**Figure 4:**
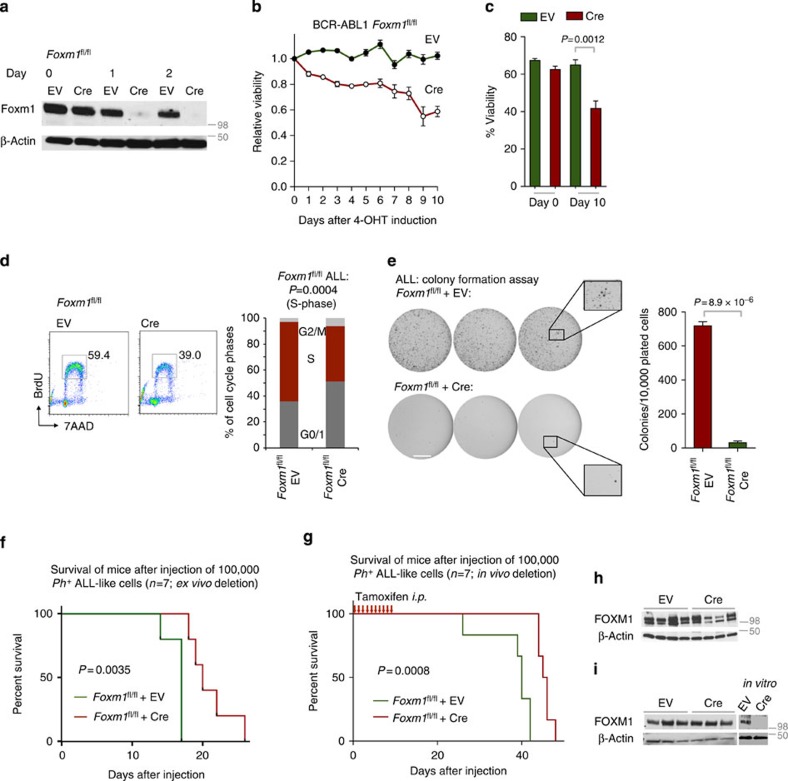
Foxm1 mediates the proliferation and survival of leukaemia cells in a mouse model of Ph^+^ ALL. (**a**) Deletion of *Foxm1* in *Foxm1*^fl/fl^ ALL cells is shown by immunoblot with β-actin as a loading control. (**b**) Viability of ALL cells after induction of deletion was measured daily by flow cytometry (fluorescence-activated cell sorting blots are shown in Supplementary Fig. 4a; representative example of three independent experiments). (**c**) Comparison of viability in *Foxm1*^fl/fl^ EV and Cre on day 0 and day 10 after 4-OHT, *n*=3; ±s.e.m., Student’s *t*-test was used for statistical analysis. (**d**) Cell cycle progression was measured based on BrdU incorporation and DNA content (7 AAD) after 2 days of 4-OHT treatment, *n*=3 (technical replicates); ±s.e.m., Student’s *t*-test was used for statistical analysis; representative example of four independent experiments. (**e**) 20,000 *Foxm1*^fl/fl^ EV and Cre ALL cells were plated in methylcellulose for 20 days in the presence of 4-OHT, colonies formed were counted; *n*=3; ±s.e.m., Student’s *t*-test was used for statistical analysis, scale bar represents 1 cm, squared section is twofold enlarged. (**f**) Kaplan–Meier analysis of NOD/SCID mice that were injected with 100,000 *Foxm1*^fl/fl^ EV or Cre ALL cells pretreated with 4-OHT for 24 h *in vitro* is shown. Statistical analysis was performed by Log-rank Mantle–Cox test. (**g**) Kaplan–Meier analysis of NOD/SCID mice that were injected with 100,000 *Foxm1*^fl/fl^ EV or Cre ALL cells and treated for 10 days with tamoxifen in corn oil i.p. is shown, *n*=7 per group. Statistical analysis was performed by Log-rank Mantle–Cox test. (**h**,**i**) Immunoblot revealed normal levels of Foxm1 after *ex vivo* and *in vivo* deletion (indicating outgrowth of clones with incomplete deletion); complete deletion *in vitro* in pre-B cells in the presence of 4-OHT is shown for day 14 (**i**).

To further analyse the function and regulation of FOXM1 in pre-B ALL cells, we focused our analysis on *BCR-ABL1*-driven ALL as a model for high-risk ALL and transformed B-cell precursors from *Foxm1*^fl/fl^ mice with BCR-ABL1 and then transduced these with a 4-OHT-inducible Cre (Cre-ER^T2^) or an empty vector (EV; ER^T2^). Immunoblot analysis revealed a rapid deletion of *Foxm1* after 4-OHT treatment (Fig. 4a), which induced cell death in a fraction of BCR-ABL1+, ALL cells (Fig. 4b,c; flow cytometric analysis is shown in Supplementary Fig. 4a). To rule out unspecific effects of Cre-mediated deletion in murine ALL, we also transduced *Foxm1*^*+/+*^ cells with EV and Cre and followed survival without observing any effect (Supplementary Fig. 4b). However, deletion of *Foxm1* resulted in arrest in both G_0_/G_1_ and G_2_/M phase with a significant reduction of the S-phase of the cell cycle (Fig. 4d) and colony formation *in vitro* was nearly entirely abolished by deletion of *Foxm1* (Fig. 4e). To investigate whether Cre-mediated deletion of *Foxm1* affects the course of leukaemia development *in vivo*, we injected 100,000 leukaemia cells carrying 4-OHT-inducible *Cre* or an empty vector control that were pretreated with 4-OHT *in vitro* for 24 h intrafemorally into sublethally irradiated NOD/SCID mice. Inducible deletion of *Foxm1* significantly prolonged survival as shown in the Kaplan–Meier survival curve (Fig. 4f; *n*=7 per group; *P*=0.0035; Log-rank Mantle–Cox test), without alteration of the ALL phenotype (representative phenotype shown in Supplementary Fig. 4c). Although we observed complete deletion of *Foxm1 in vitro* (Fig. 4a), *ex vivo* deletion and expansion of cell numbers *in vivo* revealed high levels of Foxm1 by the time of BM harvest from leukaemia-bearing mice, implying proliferative advantage of cells that evaded deletion of *Foxm1* (Fig. 4h). In addition, we deleted *Foxm1 in vivo* by treating mice with tamoxifen after injection of 100,000 leukaemia cells carrying tamoxifen-inducible *Cre* or an empty vector control. We observed that deletion of *Foxm1* significantly prolonged survival (Fig. 4g). However, similar to *ex vivo* deletion, clones that escaped deletion outgrew and formed lethal leukaemia (Fig. 4i). The presence of the *floxed* allele in these cells is confirmed by PCR, shown in Supplementary Fig. 4d. Collectively, these findings indicate that Foxm1 plays a critical role in driving ALL cells proliferation, survival and leukaemia progression. The selective outgrowth of clones that escaped Cre-mediated deletion of Foxm1 further underscores the relevance of Foxm1 in the development of fatal ALL *in vivo*.

### Foxm1 mediates TKI resistance by inducing Cat in ALL

**Figure 5:**
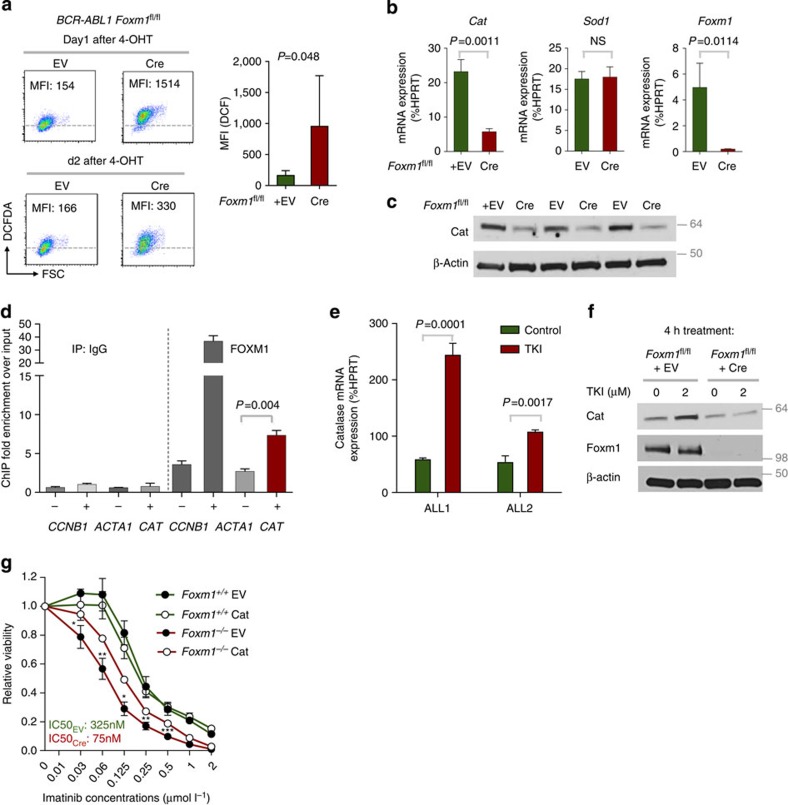
Foxm1 enables resistance to TKIs in Ph^+^ ALL. (**a**) *Foxm1*^fl/fl^ EV or Cre ALL cells were stained with 2′7′-dichlorofluorescein diacetate (DCFDA), which labels cells based on intracellular levels of ROS after the indicated time of 4-OHT treatment. Mean fluorescence intensities (MFI) for DCFDA are depicted; *n*=3 (biological replicates); ±s.e.m., Student’s *t*-test was used for statistical analysis. (**b**) qRT–PCR analysis for *Cat*, *Sod1* and *Foxm1* mRNA levels in *Foxm1*^fl/fl^ EV or Cre ALL-like cells, *Hprt* is used as a reference, *n*=3; ±s.e.m., Student’s *t*-test was used for statistical analysis. (**c**) Immunoblot analysis of Cat d2 after 4-OHT in *Foxm1*^fl/fl^ EV or Cre ALL cells is shown. β-Actin serves as a loading control; samples are derived from three independent experiments. (**d**) Single-locus ChIP assay was performed on patient-derived ALL (PDX2) with normal rabbit IgG as a negative control. (+) indicates a previously described binding domain of FOXM1, (−) indicates the negative control, either within the same gene promoter or in the *ACTA1* gene promoter; representative result of two independent experiments is shown. (**e**) *Cat* mRNA levels are measured after 4 h of imatinib treatment by quantitative real-time PCR with *Hprt* as a reference gene *n*=3; ±s.e.m., Student’s *t*-test was used for statistical analysis, two independent experiments are shown. (**f**) Cat expression levels are shown in the presence and absence of Foxm1, with and without imatinib treatment. (**g**) Imatinib (TKI)-sensitivity of BCR-ABL1-transformed *Foxm1*^fl/fl^ ALL cells was measured in dose–response experiments after 72 h, starting 2 days after 4-OHT treatment. Relative values to untreated cells are displayed. *Foxm1*^−*/*−^ and *Foxm1*^*+/+*^ cells (EV; closed circles) are shown as well as overexpression of Cat for both conditions (open circles); representative result of three independent experiments is shown.

As Foxm1 is a critical regulator of oxidative responses^6^, we analysed the intracellular reactive oxygen species (ROS) formation in the presence and absence of *Foxm1* in BCR-ABL1-transformed ALL cells and observed consistently higher ROS levels after *Foxm1* deletion (Fig. 5a) but not in Cre-transduced *Foxm1*^*+/+*^ cells (Supplementary Fig. 5a). To elucidate the underlying mechanism of this effect, we analysed the mRNA expression of the several ROS scavengers described as Foxm1 targets by qRT–PCR^6^ and found significantly reduced mRNA (Fig. 5b) and protein levels (Fig. 5c) of Cat after inducible Cre-mediated *Foxm1* deletion. Cat rapidly degrades H_2_O_2_ to O_2_ and H_2_O and therefore helps to prevent oxidative damage^38^. To verify direct CAT regulation by FOXM1 in patient-derived *Ph*^*+*^ ALL, we performed single-locus chromatin immunoprecipitation (ChIP) analysis of a known binding site of FOXM1 in intron 1 of the *CAT* gene^6^. This revealed specific binding of FOXM1, albeit low ChIP enrichment compared with the positive control *CCNB1* (Fig. 5d)^1^,^6^. The *ACTA* gene served as a negative control as well as an unrelated region in the *CCNB1* gene promoter.

Interestingly, we found that Cat expression is increased in ALL cells after TKI treatment at the mRNA and protein levels (Fig. 5e,f). This suggests that low-dose TKI treatment may initially induce ROS formation, comparable to ROS induction following growth factor deprivation^39^, which usually is compensated by a functional antioxidant response machinery, for example, by Cat expression. To evaluate whether the observed Cat upregulation by TKI is Foxm1 dependent, we treated ALL cells with TKI and evaluated Cat expression in the presence and absence of *Foxm1*. Only the Foxm1-expressing ALL cells (*Foxm1*^fl/fl^ +EV) were capable of upregulating Cat after 4 h TKI treatment (Fig. 5f). On the basis of these findings and the observed selective advantage of FOXM1-expressing cells in the presence of TKI we had observed (Fig. 2e,f), we further investigated the function of FOXM1 in mediating TKI resistance and determined TKI responsiveness of *BCR-ABL1*-driven ALL cells in the presence and absence of Foxm1. On deletion of *Foxm1*, ALL cells revealed a strikingly higher sensitivity towards TKI treatment, particularly in response to treatment with very low TKI doses. For *Foxm1*^fl/fl^ ALL cells, an IC_50_ of 325 nmol l^−1^ was measured for imatinib compared with an IC_50_ of 75 nmol l^−1^ for *Foxm1*^−/−^ ALL cells (Fig. 5g, closed circles). This was verified by Annexin V staining in the presence and absence of imatinib, with and without *Foxm1* deletion, respectively (Supplementary Fig. 5b). To define whether enforced expression of Cat can rescue the effect of *Foxm1* deletion, we overexpressed Cat in wt and *Foxm1*^−/−^ cells and conducted TKI sensitivity analysis. While Cat overexpression did not alter TKI sensitivity in the presence of Foxm1, *Foxm1*-deficient cells were significantly less sensitive in the presence of Cat as compared with EV control (Fig. 5g, open circles). Although, similar to FOXM1, FOXO3a may drive Cat transcription in the presence of TKI, we did not observe alterations in Cat expression in *Foxo3a* deficient ALL treated with TKI (Supplementary Fig. 5c)^40^.

### FOXM1 inhibition induces apoptosis and TKI sensitivity

Our genetic experiments indicate that FOXM1 is particularly relevant for proliferation and survival of leukaemic B lymphoblasts but not normal B-cell development and survival and may, therefore, represent a tumour-specific therapeutic target. For this reason, we tested two complementary approaches of pharmacological inhibition of FOXM1 based on (i) an ARF-derived peptide that inhibits FOXM1 by sequestering it to the nucleolus and mediating its degradation and (ii) FOXM1 transcriptional inactivation using the thiazole antibiotic, thiostrepton.

### Strategy I FOXM1 inhibition using a p19ARF-derived peptide

**Figure 6:**
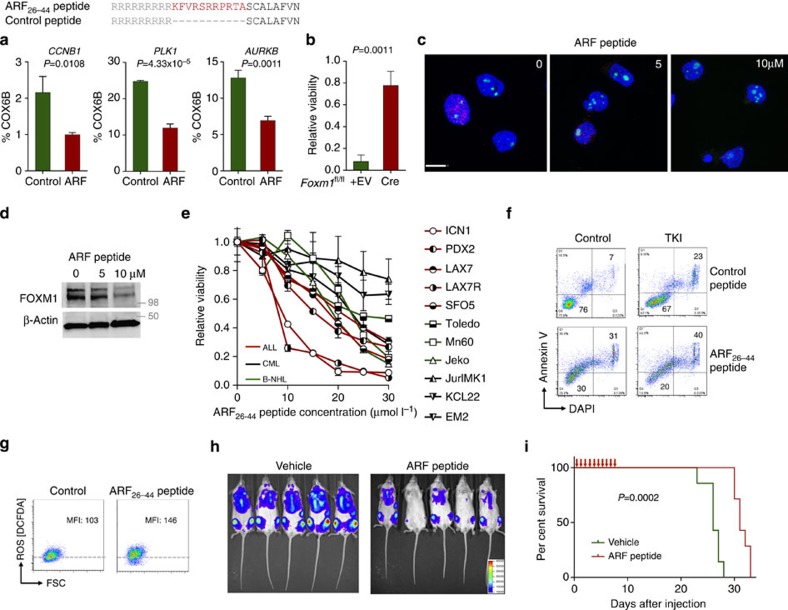
*In vivo* efficacy of a peptide-based FOXM1 inhibitor in patient-derived ALL cells. (**a**) Analysis of FOXM1 target genes after 24 h of 10 μM ARF peptide treatment of patient-derived ALL samples (shown for LAX2), COX6B was used as a reference gene, *n*=3; ±s.e.m., Student’s *t*-test was used for statistical analysis. (**b**) Efficacy of 30 μM ARF peptide on *Foxm1*^*+/+*^ and *Foxm1*^−*/*−^ cells after 72 h to confirm specificity; measured by CCK-8 viability assay; *n*=3; ±s.e.m., Student’s *t*-test was used for statistical analysis. (**c**) Confocal microscopy of control or ARF peptide-treated ALL samples (shown for LAX7R), stained for the nucleoli marker fibrillarin (green) and FOXM1 (red) and 4′,6-diamidino-2-phenylindole (DAPI). Scale bar represents 8 μm; representative result of three independent experiments. (**d**) FOXM1 protein expression after ARF peptide treatment after 24 h (shown for LAX7R) (**e**) Dose–response for ARF_26–44_ peptide of pre-B ALL versus B-NHL (green) and CML cell lines (black), ICNI and PDX2 are *Ph*^*+*^ALL, SFO5, LAX7 and LAX7R are normal karyotype ALL (red). (**f**) Annexin V/DAPI staining after 48 h of 10 μM imatinib in the presence of control peptide or ARF peptide of ICN1. (**g**) Intracellular ROS levels determined by DCFDA staining in ALL cells treated for 4 h with 10 μM ARF peptide or control peptide (PDX2). (**h**) Leukaemia burden in mice after injection of 500,000 human luciferase-labelled pre-B ALL cells (LAX7R) and treatment for 10 days with ARF peptide, measured by luciferase bioimaging. (**i**) A Kaplan–Meier analysis compared overall survival of transplant recipients in the two groups; *n*=7 per group. Statistical analysis was performed by Log-rank Mantle–Cox test.

A previously described ARF peptide binds and inhibits FOXM1 function. The tumour suppressor p19ARF directly binds to FOXM1 and has been described to mediate its translocation to the nucleolus, which may result in its degradation^10^,^36^,^41^. The p19ARF amino acids 26–44 are sufficient to mediate the inhibitory function on FOXM1 and by addition of nine lipophilic arginines, the ARF-derived peptide can permeate across the plasma membrane (ARF_26–44_)^9^,^10^. To verify the inhibitory effect on FOXM1, we treated patient-derived pre-B ALL cells with ARF_26–44_ and control peptide and analysed FOXM1 target gene transcription by qRT–PCR. As shown in Fig. 6a, we found significant inhibition of FOXM1 transcriptional activity after ARF peptide treatment. To further confirm the specificity, we treated *Foxm1*^−*/*−^ cells with ARF peptide concentrations up to 30 μM and found cells still unresponsive (Fig. 6b). To verify the described mechanism of FOXM1 translocation induced by the ARF peptide, we performed immunostaining of nucleoli with Fibrillarin and co-stained with FOXM1. Despite the observed downregulation of FOXM1 target gene, we were not able to visualize the ARF-mediated nucleolar localization of FOXM1 (Fig. 6c). However, in agreement with previous reports of ARF-targeted proteins that undergo nucleolar degradation, we observed the downregulation of FOXM1 protein levels in the presence on ARF peptide as a mechanism for FOXM1 inhibition (Fig. 6d)^42^,^43^. To confirm the finding of apoptosis induction in our genetic deletion studies of Foxm1, we treated patient-derived ALL samples with various amounts of ARF peptide and found significant growth inhibition after 72 h (Fig. 6e). B-cell lymphoma and myeloid lineage CML cells were treated in parallel experiments and were significantly less sensitive towards the ARF_26–44_ peptide. In addition, we confirmed that inhibition of FOXM1 by the ARF_26–44_ peptide sensitized patient-derived *Ph*^*+*^ ALL cells towards TKI treatment compared with TKI treatment alone (Fig. 6f) and increased intracellular levels of ROS (Fig. 6g). To evaluate the effects of the ARF peptide on leukaemic cell growth *in vivo*, we injected 500,000 luciferase-labelled, patient-derived pre-B ALL cells into NSG mice and treated the mice with 10 mg kg^−1^ ARF peptide on 10 consecutive days intravenously (i.v.) and intraperitoneally (i.p.), respectively, or vehicle control. This reduced the leukaemia burden as measured by luciferase bioimaging (Fig. 6h) and prolonged OS (Fig. 6i). Thus, the treatment results of patient-derived pre- B ALL samples with the ARF_26–44_ peptide (Fig. 6) confirmed our findings from the genetic deletion experiments in the ALL mouse model (Fig. 5) and demonstrate that peptide-based inhibition/degradation of FOXM1 protein induces selective toxicity in B-cell lineage ALL cells.

### Strategy II transcriptional FOXM1 inhibition via thiostrepton

**Figure 7:**
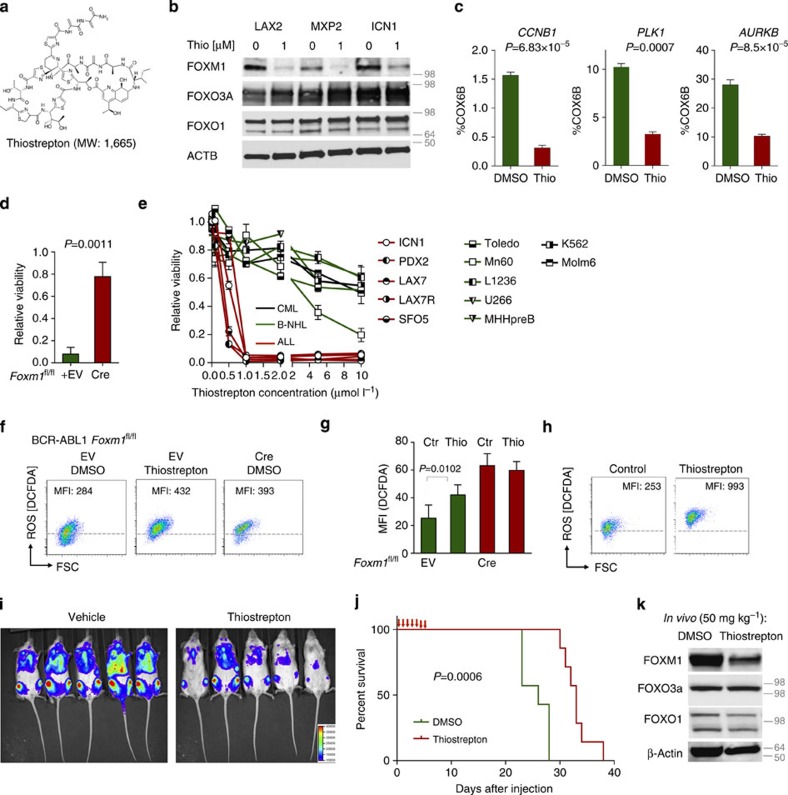
*In vivo* efficacy of the FOXM1 inhibitor Thiostrepton in patient-derived ALL cells. (**a**) Thiostrepton structure is shown. (**b**) Immunoblot of FOXM1, FOXO3a, FOXO1 and β-actin as loading control after 18 h of treatment with thiostrepton at the indicated concentration. (**c**) Analysis of FOXM1 target genes after 24 h of 10 μM ARF peptide treatment of patient-derived ALL samples (shown for LAX2). (**d**) Efficacy of 0.5 μM Thiostrepton on *Foxm1*^*+/+*^ and *Foxm1*^−*/*−^ cells after 72 h to confirm specificity; measured by CCK-8 viability assay, *n*=3; ±s.e.m., Student’s *t*-test was used for statistical analysis; representative result of three independent experiments. (**e**) Dose–response of pre-B ALL (red) versus B-cell lymphoma (green) or CML cell lines (black). (**f**) Intracellular ROS levels in *Foxm1*^fl/fl^ EV+DMSO (vehicle) and *Foxm1*^fl/fl^ EV+thiostrepton after 4 h. As a positive control *Foxm1*^fl/fl^ Cre-ER 4-OHT ALL cells (day 1) was included. (**g**) ROS levels were determined in the presence and absence of Foxm1 and with and without thiostrepton for 4 h with no further increase of ROS in the three independent Foxm1-deleted ALL-like cell lines, MFI levels are shown, *n*=3; ±s.e.m., Student’s *t*-test was used for statistical analysis. (**h**) ROS induction in human patient-derived *Ph*^*+*^ALL cells (shown for PDX2) after 4 h of thiostrepton treatment is shown. (**i**) Leukaemia burden in mice after injection of 500,000 human luciferase-labelled pre-B ALL cells (LAX7R) and treated for seven consecutive days with 50 mg kg^−1^ Thiostrepton i.v., measured by luciferase bioimaging. (**j**) A Kaplan–Meier analysis compared the OS of transplant recipients in the two groups. Statistical analysis was performed by Log-rank Mantle–Cox test. (**k**) Immunoblot analysis of FOXM1, FOXO3a and FOXO1 in human xenografted ALL (LAX7R) harvested from mice showing signs of leukaemia, and then treated with three doses of thiostrepton at 50 mg kg^−1^ or vehicle control.

In a complementary approach, we evaluated the naturally occurring antibiotic thiostrepton (structure shown in Fig. 7a), which prevents FOXM1 from binding and activating its own promoter^44^. This autoregulatory positive feedback loop is critical for sustained FOXM1 expression levels^45^,^46^,^47^,^48^. Therefore, we tested whether thiostrepton was effective in suppressing FOXM1 protein levels in patient-derived ALL cells and reduction of FOXM1 target gene expression. Importantly, thiostrepton effectively disrupted the FOXM1’s autoregulatory positive feedback loop and drastically attenuated FOXM1 expression, while the expression of other Forkhead box family members is not affected (Fig. 7b). The FOXM1 target genes *CCNB1*, *PLK1* and *AURKB* mRNA levels were significantly reduced after Thiostrepton administration, confirming its reduced transcriptional activity (Fig. 7c). In addition, we treated *Foxm1*^−*/*−^ cells with Thiostrepton and observed these mainly unresponsive up to 0.5 μM treatment for 72 h, confirming its specificity towards FOXM1 at this concentration (Fig. 7d). We then tested whether the suppression of FOXM1 induced leukaemia-specific cell death in patient-derived pre-B ALL cells and observed significant cytotoxicity at concentrations of 0.5 μmol l^−1^ thiostrepton. By contrast, B-cell lymphoma and myeloid leukaemia cells were not responsive to thiostrepton treatment at similar concentrations (Fig. 7e). In accordance with similar levels of FOXM1 expression (Supplementary Fig. 3b), we did not observe differences in Thiostrepton sensitivity between pre-B ALL subsets in regards to childhood or adult ALL and *Ph*^*+*^ or other ALL, respectively (Supplementary Fig. 5d). In addition, treatment with thiostrepton resulted in ROS accumulation and Cat regulation similar to what we had observed in the genetic deletion model of *Foxm1*: we treated *Foxm1*-expressing cells with thiostrepton for 4 h and analysed intracellular ROS levels in comparison with *Foxm1* deletion (24 h after 4-OHT). Similar increases of ROS were observed in the thiostrepton-treated *Foxm1*-expressing cells as in the *Foxm1*^−*/*−^ ALL cells (Fig. 7f). To confirm the specific role of Foxm1 in the increase of ROS formation, we included *Foxm1*^−*/*−^ ALL cells in the analysis. Thiostrepton treatment increased the formation of ROS only in the presence of Foxm1, while the ROS levels in *Foxm1*^−*/*−^ cells were not increased further by thiostrepton (Fig. 7g, representative example shown in Supplementary Fig. 5e). We subsequently verified Thiostrepton-mediated ROS formation in patient-derived *Ph*^*+*^ ALL after 4 h of treatment (Fig. 7h). To evaluate the effects of Thiostrepton *in vivo*, we injected 500,000 luciferase-labelled patient-derived pre-B ALL into NSG mice and treated the mice seven consecutive days i.v. with 50 mg kg^−1^ Thiostrepton daily or vehicle control. The daily treatment with Thiostrepton for 7 days reduced the leukaemia burden as measured by luciferase bioimaging (Fig. 7i) and prolonged OS (Fig. 7j). At the time point of harvest, the human origin of cells was confirmed by flow cytometry (representative example shown in Supplementary Fig. 5f). Confirming its biological activity *in vivo*, three doses of 50 mg kg^−1^ Thiostrepton i.v. effectively downregulated FOXM1 in xenografted ALL *in vivo* while it did not affect the expression of forkhead box transcription family members FOXO1 and FOXO3a (Fig. 7k).

## Discussion

In this study, we have identified a critical function of the transcription factor FOXM1 in mediating proliferation and drug resistance in B-cell lineage ALL, but not in normal B-cell progenitors. Using two complementary approaches based on transcriptional inactivation and peptide-based inhibition of FOXM1, we demonstrate the feasibility and efficacy of FOXM1 inhibition in ALL.

Our data indicate that besides *Ph*^+^ ALL, other subtypes of ALL with constitutive activation of FOXM1 downstream of AKT (for example, owing to the oncogenic activation of the Ras–MAPK pathway) are sensitive to FOXM1 inhibition as well and validate FOXM1 as a therapeutic target in a large fraction of drug-resistant B-cell lineage ALL. As a major factor in FOXM1-mediated drug resistance, we have identified the antioxidant molecule Cat. ROS are induced by oncogenic stimulation such as BCR-ABL1 kinase activity and drive signalling and proliferation (for example, by inhibiting phosphatase activity)^49^. However, high levels of ROS also sensitizes cells to further oxidative stress that lead to mitochondrial DNA damage and lipid peroxidation that result in cell death^50^,^51^. Similar to the effects of growth factor withdrawal or STAT5 inactivation^52^,^53^, increased ROS levels on TKI treatment may contribute to TKI-mediated cell death and require immediate compensation by antioxidant molecules^54^,^55^. We have unravelled a particular role of FOXM1 in this antioxidant response as the overexpression of Cat in *Foxm1*^−*/*−^ cells partially re-establishes TKI sensitivity but does not alter sensitivity in *wt* ALL cells (Fig. 5h). However, Cat overexpression does not completely rescue the deleterious effects of *Foxm1* deletion, suggesting that additional FOXM1-mediated mechanisms are important. Therefore, FOXM1 inhibition is advantageous over substances that enhance intracellular ROS that have been previously suggested to cooperate with BCR-ABL1 inhibition^56^,^57^. Although the function of FOXM1 as a driver of antioxidant response has been described, this link to TKI resistance is novel and further studies are required to define whether this mechanism is specific to ALL or relevant in solid tumours treated with TKI as well. Despite the difficulties of targeting transcription factors by specific small molecules, recent efforts have identified a novel FOXM1 inhibitor in a library screen of 50,000 molecules^58^.

By mediating FOXM1 translocation to the nucleolus and thereafter inducing its degradation, the tumour suppressor ARF has been described as a negative regulator of FOXM1 (ref. 9). Interestingly, the gene that encodes ARF, *CDKN2A*, is frequently deleted in lymphoid, but not myeloid, BCR-ABL1-driven leukaemia. TKI treatment of *BCR-ABL1*-driven CML patients is remarkably successful in inducing durable remissions, while monotherapy in *Ph*^+^ ALL result in relapse with TKI refractory disease within a few months. Although *CDKN2A* deletions support enhanced cell survival in multiple ways (such as reduced p53 activation via Mdm2 overexpression)^59^, the lack of negative regulation of FOXM1 may also contribute to the rapid TKI resistance development in ALL but not CML. In line with this, we have observed that CML cells are less sensitive to FOXM1 inhibition.

Taken together, our study highlights the importance of FOXM1 expression in pre-B ALL. Our findings of FOXM1 expression, itself and in combination with FOXO3a, as a potential marker for risk stratification of both childhood and adult, *Ph*^*+*^ ALL provide a robust basis for further, prospective analyses of FOXM1 in clinical trials. In addition, our data highlights the role of FOXM1 in driving the antioxidant response after TKI treatment as a crucial mechanism of TKI resistance. By genetic deletion and pharmacological inhibition, we provide a rationale for FOXM1 inhibition as a potential therapeutic intervention, alone or in synergistic combinations with TKIs for pre-B ALL and putatively other kinase-driven tumor cells that rely on a functional antioxidant response machinery.

## Methods

### Statistical analysis of clinical parameters

Gene expression microarray and patient outcome data were obtained from the GEO database accession number GSE5314 of the Eastern Cooperative Oncology Group (ECOG) Clinical Trial E2993 for adult B-ALL (*n*=54)^54^, from GSE28460 of the Children’s Oncology Group Clinical Trial P9906 for children B-ALL (*n*=49 pairs)^55^, from GSE4698 of the ALL-REZ BFM 2002 trial for children B-ALL (*n*=52)^60^, from GSE1456 of the Karolinska Hospital Stockholm–Gotland breast cancer registry for breast cancer (*n*=159)^61^ and from TCGA ( https://tcga-data.nci.nih.gov/tcga/dataAccessMatrix.htm) for AML (*n*=184). The end points of the clinical data include OS, relapse-free survival, risk, time to relapse and expression levels in samples at the time of relapse versus expression levels at diagnosis (for 49 matched sample pairs from the Children’s Oncology Group).

### Pre-B and leukaemia cell culture

Patient samples (listed in Supplementary Table 1) were provided from the Departments of Hematology and Oncology, University Hospital Benjamin Franklin, Berlin, Germany and the USC Norris Comprehensive Cancer Center in compliance with Institutional Review Board regulations (approval from the Ethik-Kommission of the Charité, Campus Benjamin Franklin and the IRB of the University of Southern California Health Sciences Campus). Informed consent was obtained from all suspects. Leukaemia cells derived from BM aspirates of patients with pre-B ALL were xenografted into sublethally irradiated NOD/SCID mice via tail vein injection. After passaging, leukaemia cells were harvested and cultured on OP9 stroma cells in α minimum essential medium without ribonucleotides and deoxyribonucleotides (Invitrogen, Carlsbad, CA), supplemented with 20% fetal bovine serum (FBS), 2 mM L-glutamine, 1 mM sodium pyruvate, 100 IU ml^−1^ penicillin and 100 mg ml^−1^ streptomycin. Cell lines used in this study are listed in Supplementary Table 2. Mouse *BCR-ABL1*-transformed pre-B cells were cultured in IMDM (Invitrogen) with GlutaMAX containing 20% FBS, 100 IU ml^−1^ penicillin, 100 μg ml^−1^ streptomycin and 50 μM β-mercaptoethanol. Normal mouse pre-B cells were cultured in the presence of 10 ng ml^−1^ IL-7. Murine LSK-like cells were cultured in the presence of 10 ng ml^−1^ recombinant mouse IL-3, 25 ng ml^−1^ recombinant mouse IL-6 and 50 ng ml^−1^ recombinant mouse SCF (PeproTech).

### Retroviral constructs and transduction

Transfection of MSCV-based retroviral constructs encoding BCR-ABL1(p210)-IRES-Neo, Cre-ER^T2^-Puro, ER^T2^-Puro, Cre-GFP, Luciferase-GFP, CD90-FOXO3a(AAA; D.A. Fruman, University of California Irvine, Irvine, CA) and empty vector controls were performed using Lipofectamine 2000 (Invitrogen) with Opti-MEM media (Invitrogen). Retroviral supernatant was produced by co-transfecting HEK 293FT cells with the plasmids pHIT123 (ecotropic env) and pHIT60 (gag-pol). 293FT cells were cultured in high glucose Dulbecco’s modified Eagle’s medium (Invitrogen) with GlutaMAX containing 10% FBS, 100 IU ml^−1^ penicillin, 100 μg ml^−1^ streptomycin, 25 mM HEPES, 1 mM sodium pyruvate and 0.1 mM non-essential amino acids. Regular media were replaced after 16 h with growth media containing 10 mM sodium butyrate. After 8 h of incubation, the medium was changed back to regular growth medium. Twenty-four hours later, the virus-containing supernatants were harvested, filtered through a 0.45-μm filter and loaded by centrifugation (2,000*g*, 90 min at 32 °C) on 50 μg ml^−1^ RetroNectin (Takara, Madison, WI) coated non-tissue culture six-well plates (Costar). Approximately 2 × 10^6^ cells were added to each well and centrifuged for 20 min at 600*g*, and subsequently maintained at 37 °C at 5% CO_2_ for 48 h before harvest. For nuclear translocation of oestrogen receptor fusion proteins, 4-OHT was added at a concentration of 500 nM.

### Cell sorting

For magnetic bead sorting, peripheral blood or BM mononuclear cells were purchased from AllCells LLC and CD19-enrichment was performed by magnetic bead cell sorting according to the manufacturer’s instructions (Miltenyi Biotech). Cell sorting by flow cytometry was performed using a BD FACSAriaII (BD Biosciences, San Jose, CA). Gating strategies and analysis of the population purity is shown in Supplementary Fig. 1.

### Western blotting

After harvesting, the cells were washed twice with PBS and lysed in CelLytic MT buffer (Sigma-Aldrich) supplemented with Mini Complete protease inhibitor (Roche), 1% phosphatase inhibitor cocktail (Calbiochem) and 1 mM phenylmethylsulphonyl fluoride. After 10 min incubation on ice and centrifugation at 11,000 *g* for 10 min at 4 °C, the protein concentration was determined by Coomassie Blue Assay (Thermo Scientific). Protein samples were loaded on 4% to 20% Bis–Tris gradient gels and transferred on Nitrocellulose membranes (BioRad). The primary antibodies used are listed in Supplementary Table 3. For protein detection, the WesternBreeze immunodetection system (Invitrogen) was used and light emission was either detected by film exposure or by the BioSpectum imaging system (UPV). Original western blots are shown in Supplementary Fig. 6.

### Flow cytometry

For analysis of normal B-cell development, BM was stained with the antibodies listed in Supplementary Table 3 and analysed using a BD LSRII Fortessa (BD Biosciences, San Jose, CA). For viability determination by flow cytometry, cells were washed with PBS and resuspended in PBS with 4′,6-diamidino-2-phenylindole (Biolegend) or propidium iodide (Sigma-Aldrich) as a marker for dead cells. For detection of apoptotic cells, cells were washed twice in PBS containing 5% bovine serum albumin (BSA) and stained with Alexa-Fluor 647-labelled Annexin V, according to the manufacturer’s protocol (Biolegend). For cell cycle analysis, the BrdU Cell Proliferation Assay Kit was purchased from BD Biosciences and performed according to the manufacturer’s protocol. In brief, 1 × 10^6^ cells ml^−1^ were cultured for 1 h in the presence of 1 μM BrdU, washed with PBS, fixed and stained according to protocol and analysed by flow cytometry. For the analysis of intracellular ROS formation, ALL cells were incubated for 7 min with 500 nM 5-(and 6-)chloromethyl-2′,7′dichlorodihydrofluorescein diacetate (CM-H2DCFDA, Invitrogen) at 37 °C for oxidation of the dye by ROS. After washing with PBS, the cells were incubated for an additional 15 min at 37 °C in PBS to allow complete deacetylation of the oxidized form of CM-H2DCFDA by intracellular esterases. The levels of fluorescence were then directly analysed by flow cytometry, gating on viable cells.

### Colony-forming assay

The methylcellulose colony-forming assays were performed with 10,000 BCR-ABL1-transformed mouse pre-B ALL cells. Cells were resuspended in mouse MethoCult medium (StemCell Technologies) and cultured on 3-cm diameter dishes, with an extra water supply dish to prevent evaporation. After 21 days, colony numbers were counted by a GelCount analyzer (Oxford Optronix).

### Quantitative RT–PCR

Total RNA from cells was extracted using the RNA isolation kit from Macherey Nagel. Complementary DNA was generated with the Vilo SuperScript cDNA synthesis kit (Invitrogen). Quantitative real-time PCR was performed with Fast SYBR Green Master Mix (Invitrogen) and the Viaa7 real-time PCR system (Life Technologies) according to standard PCR conditions. Primer sequences are listed in Supplementary Table 4. For human samples, COX6B was used as a reference gene, for mouse samples Hprt.

### Cell viability assay

Imatinib (Novatis Pharmaceuticals, Basel, Switzerland) was reconstituted in distilled water at 10 mM and aliquots were stored at −20 °C. Thiostrepton was purchased from Sigma, reconstituted in dimethylsulphoxide (DMSO) to 10 mM and stored at −20 °C. The ARF- and its control peptide were dissolved in DMSO to a final concentration of 10 mM and stored at −80 °C. 5 × 10^4^ ALL cells were seeded on 96-well plate and drugs were added at indicated concentrations. After 3 days, 15 μl of Resazurin (AbD Serotech) were added and cells were incubated for 4 h before the emission was read at 535 nm using a Spectramax plate reader (Molecular devices). The fold changes were calculated by using baseline values of untreated cells as a reference.

### Single-locus ChIP

Single-locus ChIP was performed as described^62^. In brief, 1 × 10^8^ cells were crosslinked with 1% formaldehyde for the indicated conditions, and then lysed in 1 ml lysis buffer, and sonicated with six rounds of 30′′ on/off, 5 min each on a bioruptor (Diagenode). The sonication efficacy was determined by resolution on an agarose gel after de-crosslinking. After preclearing with Protein A Agarose (Invitrogen), 5 μg per sample FOXM1 antibody (C20-X; Santa Cruz) or normal rabbit IgG (Santa Cruz) was added for overnight (ON) incubation. Then 50 μl of 50% Protein A Agarose slurry was added for 1 h, followed by washing with low salt, high-salt wash buffer and lithium chloride buffer. The DNA was decrosslinked in the presence of 0.2 M NaCl at 65 °C ON. RNA was digested and DNA was purified with the Macherey Nagel PCR purification kit. PCR was performed as described in the section for quantitative real-time PCR. A list of primers is provided in Supplementary Table 4.

### Immunofluorescence analysis

Cells were seeded onto retronectin-coated chamber slides and treated with 5 and 10 μM ARF peptide for 18 h. Cells were fixed with 4% paraformaldehyde for 10 min at room temperature (RT), permeabilized in PBS/0.15% Triton-X-100 for 2 min at RT and blocked in 1% BSA/PBS for 1 h at RT. Slides were then incubated with antibodies against fibrillarin (clone 38F3, ab4566, 1/250) and FOXM1 (clone D12D5, CST#5436, 1/50) in 1% BSA/PBS ON at 4 °C. Slides were washed three times for 5 min in PBS and incubated for 1 h at RT in 1% BSA/PBS with Alexa Fluor 488-conjugated goat anti-mouse (Molecular Probes, A-11029) and Alexa Fluor 594-conjugated goat anti-rabbit (Molecular Probes, A-11037) secondary antibodies. Slides were then washed three times for 5 min in PBS and mounted using Vectashield (Vector Laboratories) containing 1 μg ml^−1^ 4′,6-diamidino-2-phenylindole. Images were acquired on a Leica SP5 upright confocal microscope and processed using Volocity software (Perkin Elmer).

### *In vivo* leukaemia cell transplantation

All mouse experiments were subject to institutional approval by the University of California San Francisco Institutional Animal Care and Use Committee. BCR-ABL1-transformed murine *Foxm1*^fl/fl^ pre-B ALL cells transduced with either empty vector (EV, ER^T2^ without Cre) or Cre (Cre-ER^T2^) containing a puromycin resistance cassette. After selection for 2 days with 2 μg ml^−1^ puromycin, successful selection was verified by fluorescence-activated cell sorting analysis (no living cell in untransduced control cells) and subsequently grown *in vitro* for expansion of cell numbers. Experiments were performed within 4 weeks after BM harvest to avoid secondary mutations. *Ex vivo* deletion of *Foxm1* was induced in the Cre-ER^T2^ group by adding 4-OHT 24 h before transplantation. To rule out the possible homing defects after Foxm1 deletion as a potential confounding variable, cells were injected intrafemorally into sublethally irradiated (2.5 Gy) NOD/SCID mice. Seven mice per group were injected. For *in vivo* deletion of Foxm1, 100,000 BCR-ABL1-transformed murine *Foxm1*^fl/fl^ pre-B ALL cells with either EV or Cre were injected *i.v*. and deletion induced by tamoxifen injection daily at 0.4 mg per mouse for 10 days. Terminally ill mice were killed and spleen and/or BM harvested for further analysis. For treatment of human xenografts, LAX7R cells were transduced with a luc-GFP vector and sorted for GFP expression. Cells (5 × 10^5^) were injected i.v. into NSG mice (NOD/SCID-IL2 receptor gamma chain knockout), seven mice per group and treatment was initiated. ARF peptide was administered i.v. and i.p. at 10 mg kg^−1^ daily (total dose 20 mg kg^−1^) for 10 consecutive days and Thiostrepton i.v. at 50 mg kg^−1^ daily for seven consecutive days. ARF peptide was resolved fresh every day to avoid freeze-thawing steps. C-terminal amidation and N-terminal acetylation was used to increase peptide half-life *in vivo*. Thiostrepton was resolved at 25 mg ml^−1^ in DMSO and diluted in PBS before injection. To increase solubility, 2 min of sonication was performed daily before injection. Bioimaging of leukaemia burden in the mice was performed at day 14 using an *in vivo* IVIS 100 bioluminescence/optical imaging system (Xenogen). D-Luciferin (Promega) was injected intraperitoneally at a dose of 2.5 mg per mouse in PBS 12 min before measuring the luminescence signal. General anaesthesia was induced with 5% isoflurane and continued during the procedure with 2% isoflurane introduced through a nose cone. When a mouse became terminally sick, it was killed, and BM and/or spleen were collected for further analysis.

## Additional information

**How to cite this article:** Buchner, M. *et al*. Identification of FOXM1 as a therapeutic target in B-cell lineage acute lymphoblastic leukaemia. *Nat. Commun*. 6:6471 doi: 10.1038/ncomms7471 (2015).

## Supplementary Material

Supplementary Information Supplementary Figures 1-6 and Supplementary Tables 1-4
