# Supplementary material for: Identification of FOXM1 as a therapeutic target in B-cell lineage acute lymphoblastic leukaemia
Source: Nat Commun. 2015 Mar 10;6:6471. doi: 10.1038/ncomms7471 (PMC4366523; doi:10.1038/ncomms7471)
Supplement: Supplementary Information — Supplementary Figures 1-6 and Supplementary Tables 1-4 [file ncomms7471-s1.pdf]

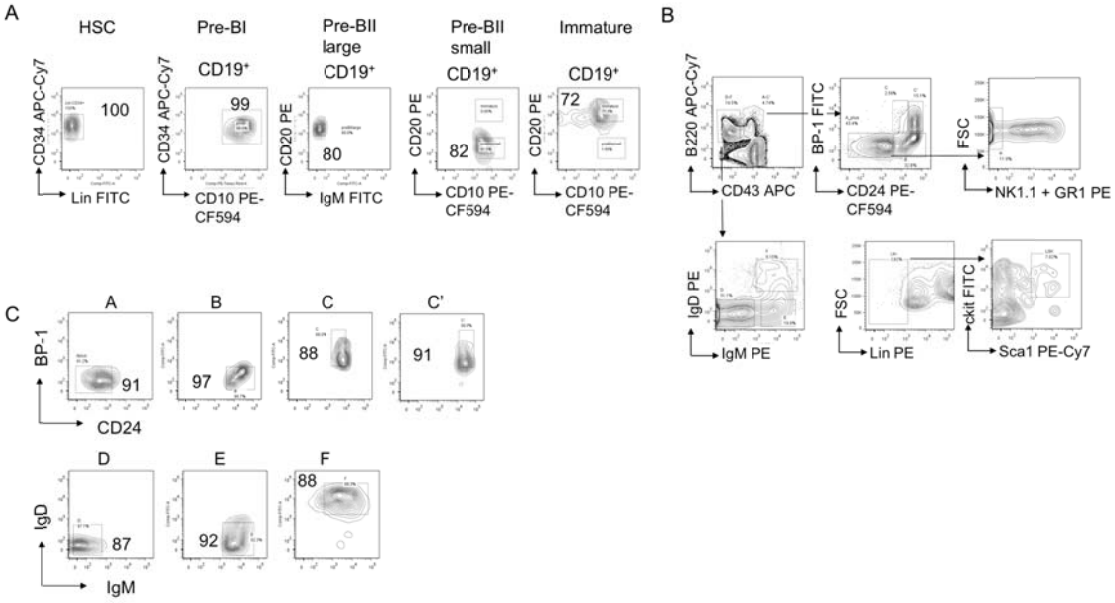

Supplementary Figure 1: *Foxm1 expression in early B cell development-sorting strategy and verification*

(A) BM from a healthy donor was stained and sorted as described in (van Zelm *et al*., 2005), reanalysis after the sort of the different fractions is displayed, numbers indicate % purity in the displayed gate. For the pro B faction we were not able to collect sufficient amount of cells for further analysis. (B) Sorting strategy for murine B cell precursors (Hardy *et al*., 1991). BM of 4 6-weeks old C57/B6 mice were pooled and stained in 3 different tubes and sorted as displayed. (C) Reanalysis after the sort is shown for Hardy Fraction A-F, numbers indicate % purity in the displayed gate.

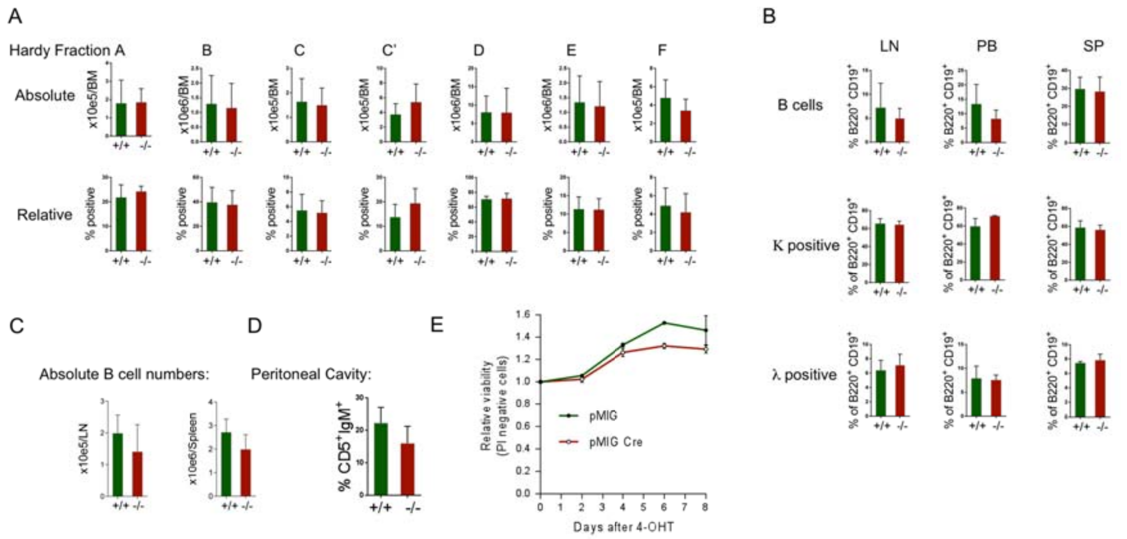

Supplementary Figure 2: *Analysis of B cell development in the absence on Foxm1*
(A) BM of 6-8 weeks old *Mb1*$^{Cre\text{-}tg/+}$ *Foxm1*$^{fl/fl}$ (n=4) and Mb1 wt (n=7) littermates were analyzed by flow cytometry according to the Hardy Fractions (scheme displayed in suppl. Figure 1B). Absolute cell count/BM and relative values of parent gate are shown. (B) Lymph node (LN), peripheral blood (PB), and spleen (SP) were analyzed for the expression of CD19, B220, κ- and λ LC. (C) Absolute B cell counts are displayed for spleen and lymph node. (D) Peritoneal cavity was analyzed for the presence of $CD5^+$ IgM^+ B1 B cells. None of the displayed analyses revealed statistical significance using student's t-test; $P>0.05$, error bars represent SEM. (E) Foxm1 deletion in LSK-like cells does not influence growth or apoptosis rate as shown by GFP competition assay.

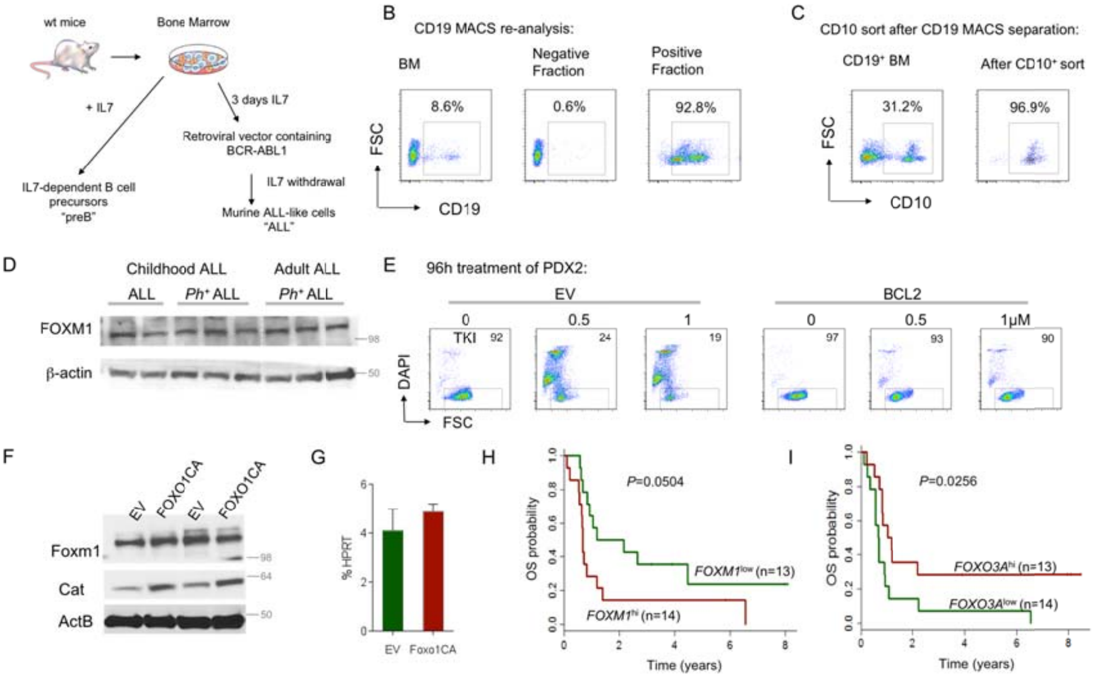

Supplementary Figure 3: *Foxm1 regulation and clinical relevance*

(A) Schematic of model used to generate murine ALL-like cells and IL-7 dependent B cell precursors. (B) Sort efficacy is shown after magnetic bead separation of $CD19^+$ B cells. (C) Re-analysis after $CD19^+$ MACS and $CD10^+$ B cell progenitor sort. (D) FOXM1 levels were compared in different ALL subtypes. Loading: SFO6R (MLLr) MXP9 (normal karyotype) MXP2 MXP4 MXP5 ICN1 PDX2 LAX2 (all Ph^+); (E) Viability EV and BCL2-overexpressed ALL cells in the presence and absence of TKI for 96h. (F) Foxm1 levels after overexpression of inducible FOXO1CA, 2days after induction with 4-OHT. Cat is shown as a positive control and actin as loading control. (G) mRNA levels of Foxm1 after FOXO1CA activation, n=3, error bars indicate SEM. (H+I) Patients with Ph^+ ALL in the ECOG E2993 trial (n=55) were segregated into four groups based on higher or lower than median mRNA levels of FOXM1 (H) and FOXO3A (I).

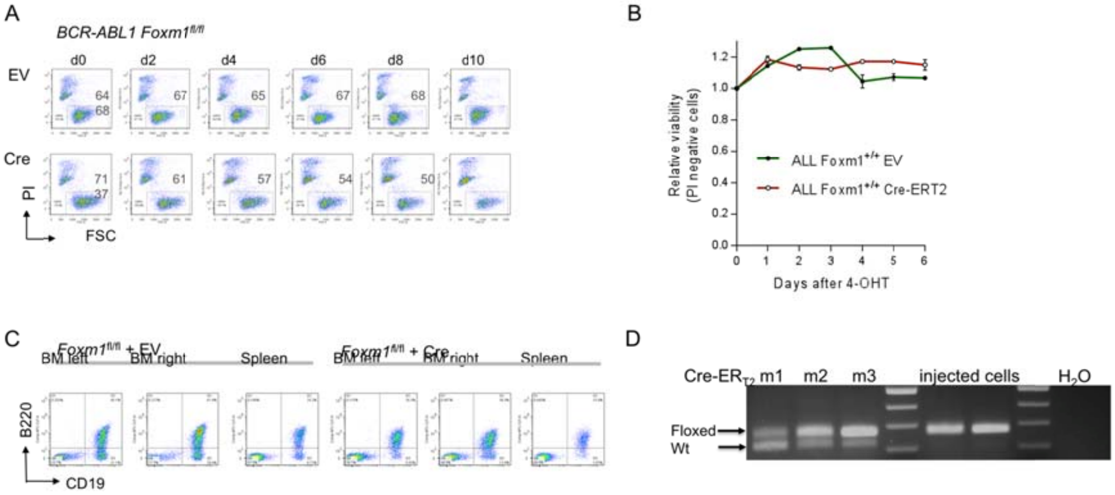

Supplementary Figure 4: *Genetic deletion and phenotypic analysis of Foxm1*$^{fl/fl}$ cells
(A) Viability measured by PI staining after induction of deletion of Foxm1 in ALL-like cells. (B) Cre activation in Foxm1+/+ cells does not influence survival of ALL cells. (C) After injection of 100,000 murine *Foxm1*$^{fl/fl}$ Cre-ERT2 or EV ALL-like cells into the right femur, BM was harvested from mice showing signs of leukemia burden (hunched back, weight loss, inability to move) and analyzed for expression of CD19 and B220. No difference was observed in the phenotype of the leukemia from *Foxm1*$^{fl/fl}$ Cre-ER^{T2} or EV, nor of the distribution (migration capacity) from right femur to left in the groups of *Foxm1*$^{fl/fl}$ Cre-ER^{T2} or EV. A representative example is shown. (D) Confirmation of presence of floxed gene locus after *in vivo* deletion of FOXM1 from unsorted splenocytes.

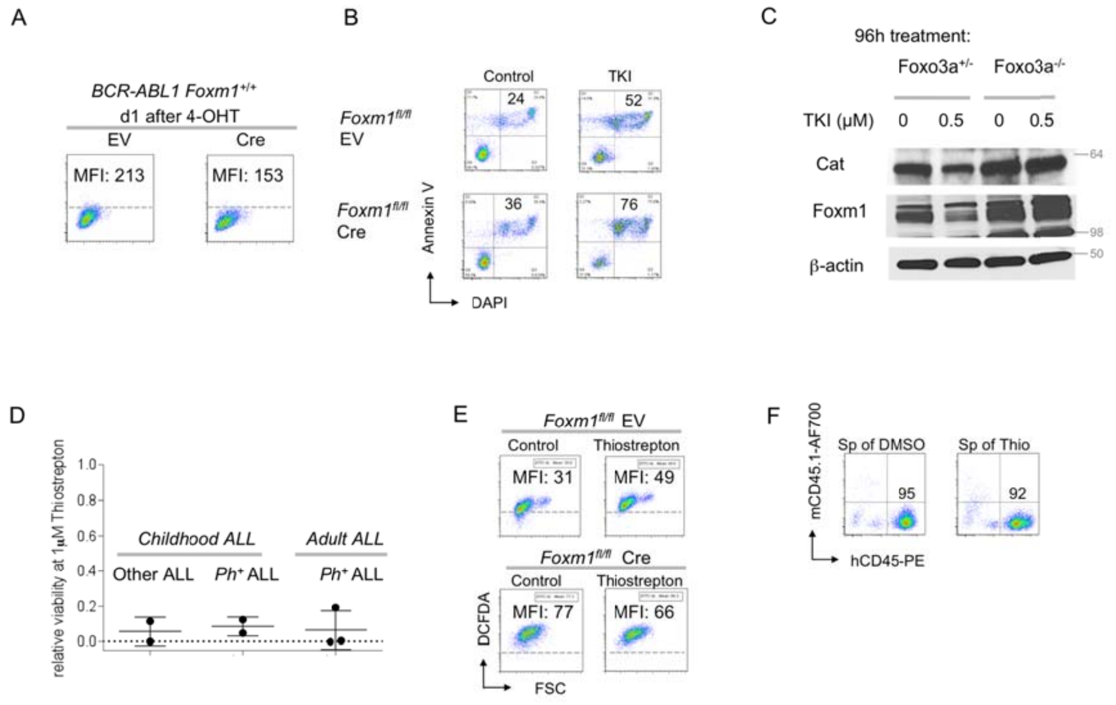

Supplementary Figure 5: *FOXM1 inhibitor specificity analysis*
(A) DCF staining after 4-OHT induction in *Foxm1*$^{+/+}$ ALL-like cells. (B) Confirmation of higher sensitivity to Imatinib in the absence of Foxm1 by Annexin V and DAPI staining. (C) Catalase expression in *Foxo3a*$^{+/-}$ and *Foxo3a*$^{-/-}$ cells in the absence and presence of TKI for 96h. (D) Thiostrepton sensitivity of *Ph*$^{+}$ and other ALL subsets of adult or childhood ALL (E) Representative DCFDA staining for intracellular ROS in the presence and absence on Foxm1, treated with and without Thiostrepton. (F) Human origin of ALL cells infiltrating the spleen was confirmed by flow cytometry.

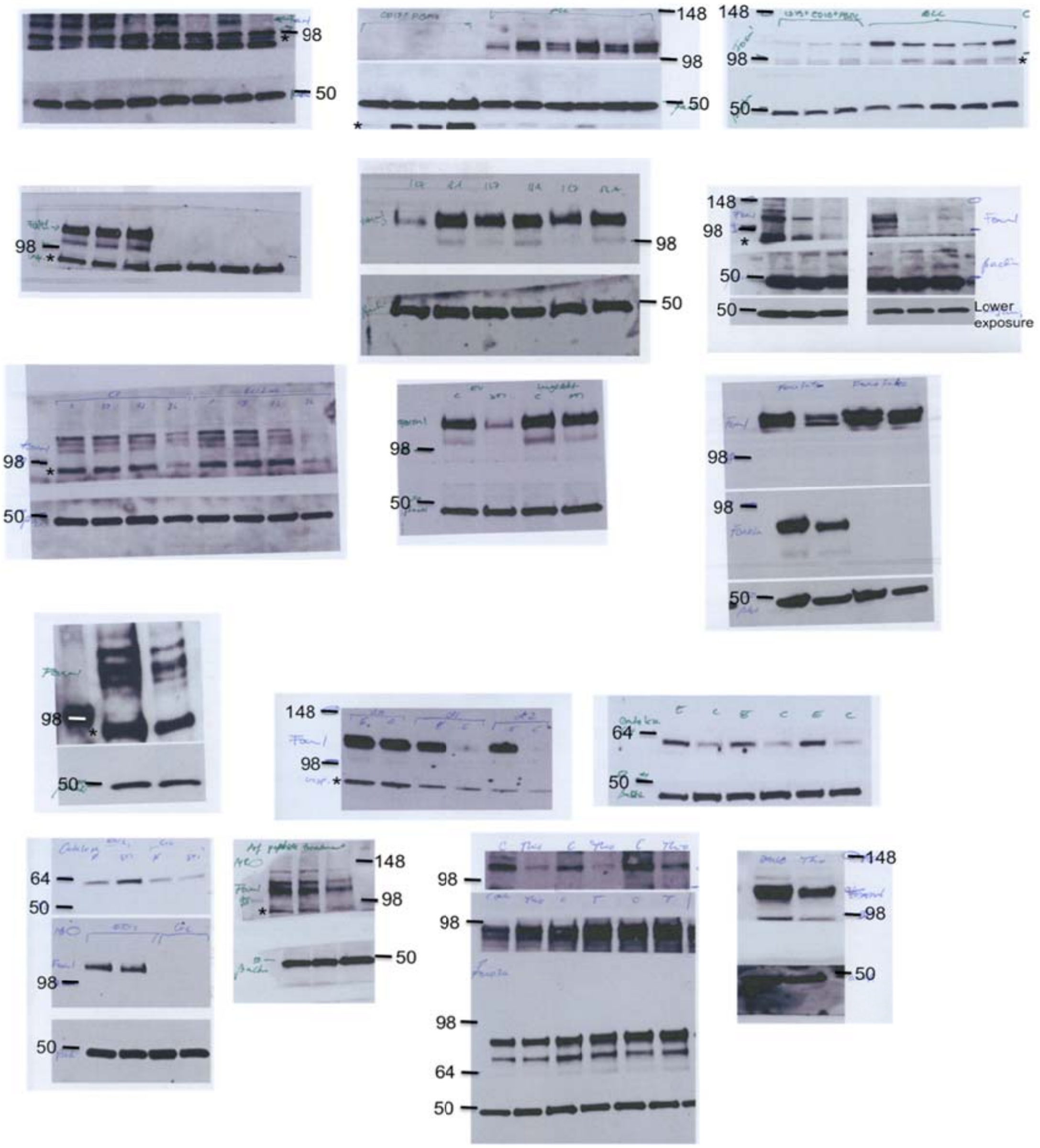

Supplementary Figure 6: *Original immunoblots*

Immunoblots are ordered as they appear in the main figures. (*) indicate unspecific bands, as determined by molecular weight of the target protein and/or knockout controls. For FOXM1, the unspecific band appears when the antibody sc-500 was used (see Supplementary Table).

Supplementary Table 1: *List of primary ALL cases*

Case	Genetic alteration(s)	Disease stage	Gender	Sample	Age
SFO6R	MLL-rearrangement	relapse	male	peripheral blood	<1
MXP9	Unknown, PAX5 deletion	unknown	unknown	bone marrow	2
MXP2	t(9;22)(q34;q11) p190	unknown	unknown	bone marrow	3
MXP4	t(9;22)(q34;q11) p210	unknown	unknown	bone marrow	13
MXP5	t(9;22)(q34;q11) p210	unknown	unknown	bone marrow	5
ICN1	t(9;22)(q34;q11)	diagnosis	male	bone marrow	14
PDX2	t(9;22)(q34;q11)	diagnosis	female	bone marrow	52
LAX2	t(9;22)(q34;q11) T315I	relapse	male	bone marrow	38
BLQ5	t(9;22)(q34;q11) T315I	relapse	female	bone marrow	unknown
SFO5	normal karyotype	diagnosis	male	peripheral blood	23
LAX7	normal karyotype	diagnosis	unknown	bone marrow	unknown
LAX7R	normal karyotype; KRasG12V	Relapse	unknown	bone marrow	unknown

Supplementary Table 2: *List of Lymphoma and CLL cell lines*

Name	Disease	Genetic alteration
U266	Myeloma	P16INK4A methylation, P53 mutation; MYC and Cyclin D1 overexpression
MHH-PREB-1	B-NHL	t(8;14) MYC-IGH alteration, hyperdiploidy
MN-60	Burkitt lymphoma	t(8;14) MYC-IGH alteration
L1236	Hodgkin lymphoma	hypotriploid karyotype with 17% polyploidy; BCL6 mutation
Toledo	DLBCL	multiple chromosomal aberrations
JURL-MK1	CML	BCR-ABL1 b3-a2; hypodiploid with 4% polyploidy
KCL22	CML	BCR-ABL1 b2-a2; hyperdiploid karyotype with 3.3% polyploidy; mutations: C/EBPA (both alleles), C/EBPB, P53
EM2	CML	BCR-ABL1 b3-a2; hypertriploid with hypotetraploid sideline; P53 mutation
Molm6	CML	BCR-ABL1; P53 mutation
K562	CML	BCR-ABL1 b3-a2; ABL amplification, P15INK4B deletion, P16INK4A deletion, P53 mutation

Supplementary Table 3: *List of antibodies*

Antibodies used for flow cytometry (anti-mouse):	
B220-BV421 and - APC-Cy7	Biolegend
CD43-APC	Biolegend
BP-1 FITC	Biolegend
NK1.1 PE and GR1-PE	Biolegend
CD19-Alexa Fluor 488 and –PE	Biolegend
IgD-FITC,–APC-Cy5.5, and APC-Cy7	Biolegend
IgM PE and –PerCP-Cy5.5	Biolegend
Lin – PE	Biolegend
ckit FITC	BD Bioscience
κ LC PE-CF594 and -PE	BD Bioscience
λ LC PE	BD Bioscience
Sca1-PE-Cy7	BD Bioscience
CD24 PE-CF594	BD Bioscience
Annexin V Alexa Fluor 647	BD Bioscience
TruStain fcX Fc block	Biolegend

Antibodies used for flow cytometry (anti-human):	
Lin(CD3/33/56)-FITC	BD Bioscience
CD34 APC-Cy7	Biolegend
CD10-PE-CF594	BD Bioscience
CD19 Qdot655	Invitrogen
CD22 PE	BD Bioscience
CD20 PE	Biolegend
IgM FITC	Biolegend
Human Fc block	Miltenyi Biotechnology

Antibodies used for immunoblot/ChIP with dilutions:			
FOXM1	Cell Signaling Technology	Cat# 3948	1:1000
FOXM1	Santa Cruz Biotechnology	Cat# sc-500	1:500
FOXM1 for ChIP	Santa Cruz Biotechnology	Cat# sc-502	5μg/1ml
Catalase	Cell Signaling Technology	Cat# 8841	1:1000
FOXO1	Cell Signaling Technology	Cat# 2880	1:1000
FOXO3a	Cell Signaling Technology	Cat# 2497	1:1000
β actin	Santa Cruz Biotechnology	Cat# sc-47778	1:5000
normal rabbit IgG	Santa Cruz Biotechnology	Cat# sc-2027	5μg/1ml

Supplementary Table 4: *List of primer sequences*

Primer sequences for single locus ChIP analysis:	
hCCNB1neg_FW	5'-GCAATCTGCTTCCACAAACTC-3'
hCCNB1neg_RV	5'-GGCTTCTCAGTTTTAGCTCACG-3'
hCCNB1pos_FW	5'-AATGGGAAGGGAGTGAGTGC-3'
hCCNB1pos _RV	5'-ACCTACACCCAGCAGAAACC-3'
hCAT_FV	5'-GATGGGCGGATTTCTTGAGG-3'
hCAT_RV	5'-AGTAGTTGGGATTACAGGCGA-3'
hACTA1_FW	5'- AGTCAGCAGTCAGGCACCTT-3'
hACTA1_RV	5'- AGTGTCTCCCTAGCGGGTTT-3'
Primer sequences for quantitative real-time PCR:	
mCatalase_FW	5'-TGAGAAGCCTAAGAACGCAATTC-3'
mCatalase-RV	5'-CCCTTCGCAGCCATGTG-3'
mSod1_FW	5'- GTGATTGGGATTGCGCAGTA -3'
mSod1-RV	5'- TGGTTTGAGGGTAGCAGATGAGT -3'
mFoxm1_FW	5'- CACTTGGATTGAGGACCACTT -3'
mFoxm1-RV	5'- GTCGTTTCTGCTGTGATTCC -3'
mHprt_FW	5'-GGGGGCTATAAGTTCTTTGC-3'
mHprt_RV	5'-TCCAACACTTCGAGAGGTCC-3'
hFOXM1_FW	5'-CACCCCAGTGCCAACCGCTACTTG-3'
hFOXM1_RV	5'-AAAGAGGAGCTATCCCCTCCTCAG-3'
hCOX6B_FW	5'- AACTACAAGACCGCCCCTTT -3'
hCOX6B_RV	5'- GCAGCCAGTTCAGATCTTCC -3'
hCCB1_FW	5'- AAGGCGAAGATCAACATGGC -3'
hCCB1	5'- TTTGGCCTGCAGTTGTTCAC -3'
hPLK1_FW	5'- ACGGCTTTTTCGAGGACAAC -3'
hPLK1_RV	5'- TGGCAGCCAAGCACAATTTG -3'
hAURKB_FW	5'- ATCTGCTCTTAGGGCCAAGGG -3'
hAURKB_RV	5'- CACATTGTCTTCCTCCTCAGGG -3'
mFoxm1fl_FW	5'- TGGCTTCCCAGCAGTACAAATC -3'
mFoxm1fl_RV	5'- TGCTTACAAAAGACACACTTGGACG -3'
